# Airplane transport isolators may loose leak tightness after rapid cabin decompression

**DOI:** 10.1186/s13049-015-0090-6

**Published:** 2015-02-08

**Authors:** Roland Albrecht, Andres Kunz, Wolfgang G Voelckel

**Affiliations:** Swiss Air Rescue, REGA, Zürich, Switzerland; Department of Anesthesiology, Kantonsspital, St. Gallen, Switzerland; Aeromedical Institute, Swiss Air Force, Dübendorf, Switzerland; Department of Anesthesiology and Critical Care Medicine, AUVA Trauma Center, Dr.-Franz-Rehrl-Platz 5, A-5010 Salzburg, Austria; ÖAMTC Austrian Air Rescue, Vienna, Austria; Innsbruck Medical University, Innsbruck, Austria; Paracelsus Private Medical University of Salzburg, Salzburg, Austria; Department of Health Studies, University of Stavanger, Stavanger, Norway

**Keywords:** Air medical transport, Infectious diseases, Decompression, Isolation chamber

## Abstract

Air medical transport of patients suffering of highly infectious diseases is typically performed employing portable isolation chambers. Although the likelihood of decompression flight emergencies is low, sustainability of the devices used is crucial. When a standard isolation unit was subjected to an explosive cabin decompression of 493 hPa, simulating a 32808 ft flight level accident, leak tightness of the unit was lost due to rupture of the bag caused by over expansion. When the pressure chamber experiment was repeated with a modified unit, distension was minimized by an additional compensation air bag, thus ensuring leak tightness.

**Electronic supplementary material:**

The online version of this article (doi:10.1186/s13049-015-0090-6) contains supplementary material, which is available to authorized users.

## To the Editor

The current Ebola epidemic highlights air transport challenges of infectious patients. The Guidance on Air Medical Transport for Patients with Ebola Virus Disease published by the Centers for Disease Control and Prevention recommend a portable isolation unit to contain infected materials and minimize contamination of the aircraft. [1] At present, air carriers employ two different devices: smaller single-person isolation units or the larger Aeromedical Biological Containment System, resembling a plastic tent. Under undisturbed flight conditions, both technologies have been shown feasible and effective in preventing contamination. When optimizing our transport protocol, we had to recognize that little is known about safety in case of flight emergencies such as rapid cabin decompression. Accordingly, we tested the effects of an explosive decompression on the sustainability of the chamber currently used in our program (VenIONPIU, TB-Safety Ltd., Frick, Switzerland).

Tests were performed at the Swiss Air Force Aeromedical Center in Dübendorf, Switzerland. In two hypobaric chambers, separated by a standard airplane window size membrane, pressures were adjusted to the typical pressure difference between in-cabin and ambient pressure at 22965, 27886, 32808 and 33464 ft flight level, respectively. To simulate an explosive decompression, the membrane was cracked in four consecutive experiments, resulting in an immediate pressure drop of 360, 424, 493 and 500 hPa (5.22, 6.15, 7.15 and 7.25PsI) within 1.5-3 seconds, respectively. The single-patient isolation unit was mounted on a stretcher as usual and monitored by video surveillance. For each of the first three tests, a new isolation unit was used, whereas in the fourth setting, reproducing a decompression scenario at 33464 ft flight level, a modified unit with an additional air bag designed to compensate volume expansion was employed.

## Findings

**Figure 1:**
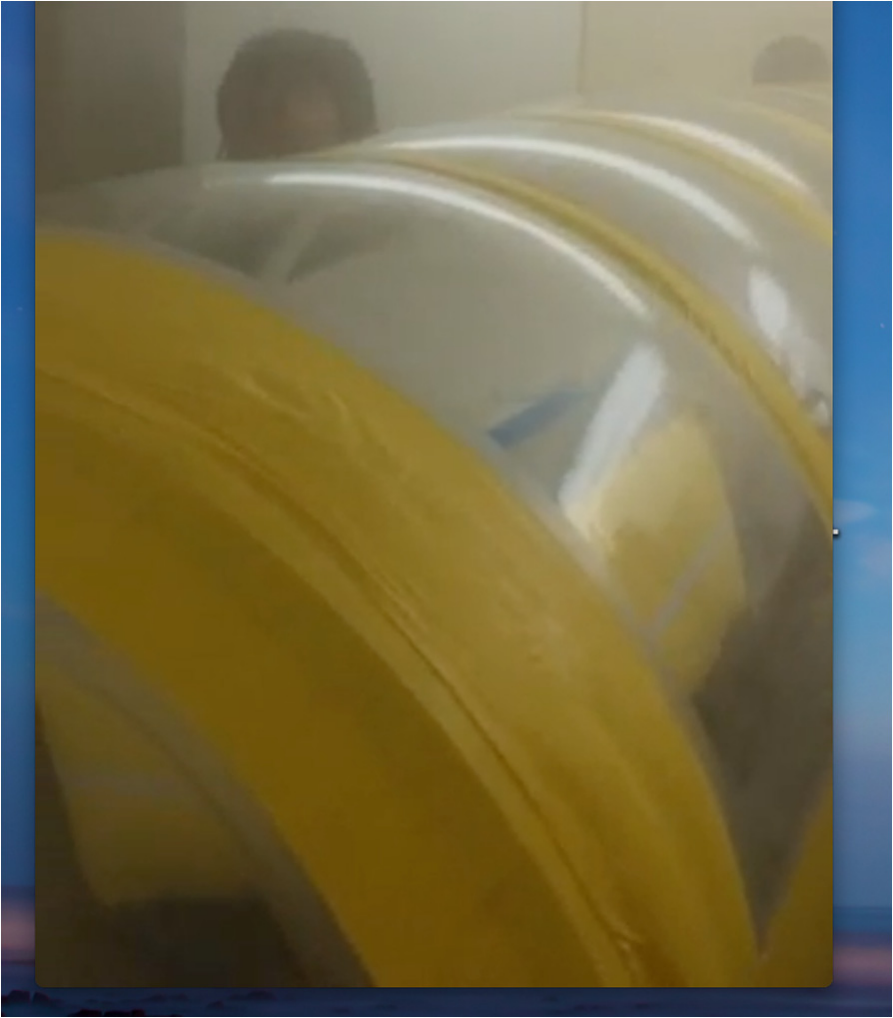
**Over expanded single-patient isolation chamber during explosive decompression.**

**Figure 2:**
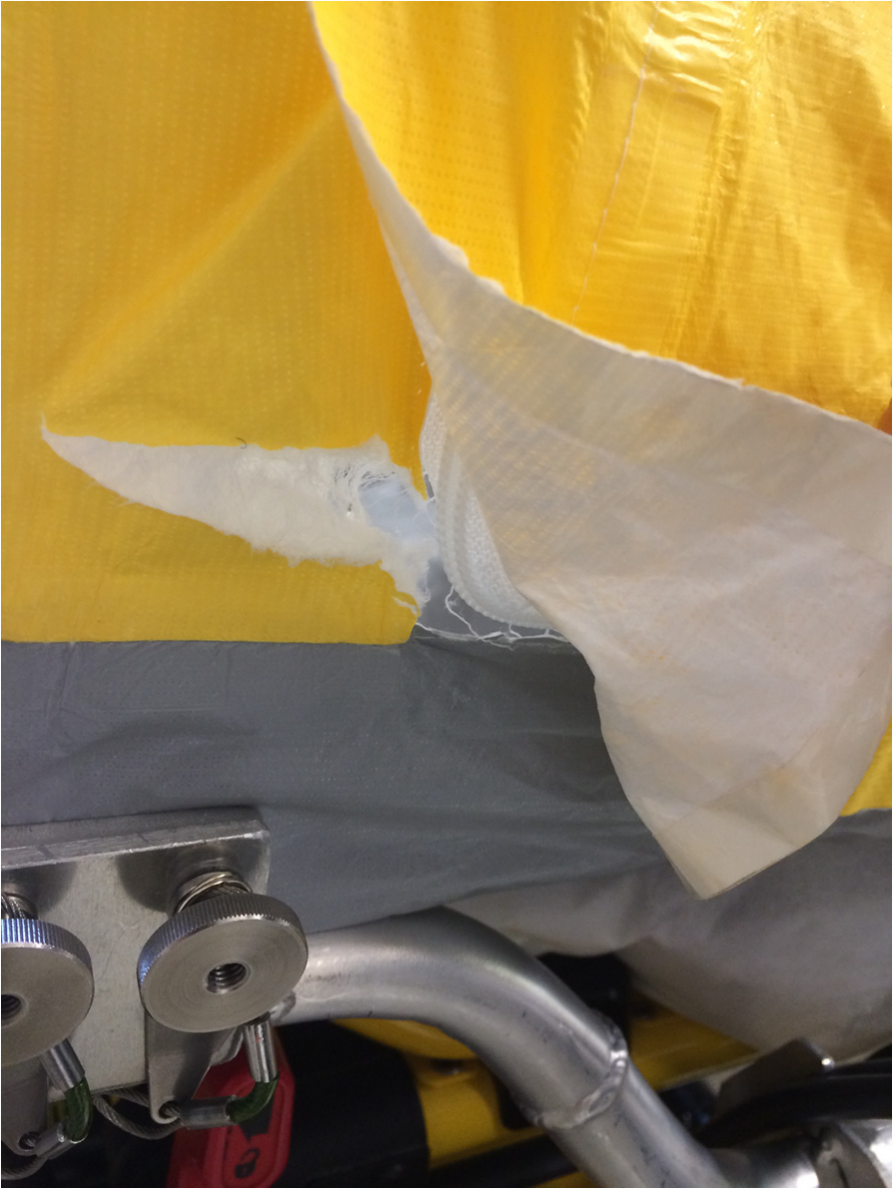
**Ruptured suture after explosive decompression (Δ p 493hPa/7.15PsI).**

As defined by the law of Boyle and Mariotte, air volume within the patient isolation unit expanded with increasing Δ p by1.6-2.1, respectively, thus causing overexpansion of the bag (Additional file 1). In the third test when Δ p was 493 hPa (7.15PsI), leak tightness of the unit was lost due to a ruptured suture (Figures 1 and 2). When the experiment was repeated with the modified unit (Δ p 500 hPa/7.25PsI), distension of the patient chamber was minor due to the compensation air bag and leak tightness was maintained.

Although incidence of flight emergencies is low, decompression tests of isolation units should be mandatory in order to guarantee safety for air transport of patients with hemorrhagic fever, SARS, asian flu and others. Leak tightness of can be improved with an additional air bag.

## Additional file

Additional file 1:
**Videoclip demonstrating overexpansion of the isolation bag during explosive decompression.**
